# Macrophage scavenger receptor A1 promotes skeletal muscle regeneration after hindlimb ischemia

**DOI:** 10.7555/JBR.38.20240117

**Published:** 2024-05-29

**Authors:** Siying Wang, Saiya Wang, Wenhan Cai, Jie Wang, Jianan Huang, Qing Yang, Hui Bai, Bin Jiang, Jingjing Ben, Hanwen Zhang, Xudong Zhu, Xiaoyu Li, Qi Chen

**Affiliations:** 1 Department of Pathophysiology, Key Laboratory of Targeted Intervention of Cardiovascular Disease and Molecular Intervention, Collaborative Innovation Center for Cardiovascular Disease Translational Medicine, Nanjing Medical University, Nanjing, Jiangsu 211166, China; 2 The Affiliated Suzhou Hospital of Nanjing Medical University, Suzhou Municipal Hospital, Gusu School, Nanjing Medical University, Nanjing, Jiangsu 211166, China

**Keywords:** scavenger receptor A1, macrophage, hindlimb ischemia, oncostatin M

## Abstract

The macrophage-mediated inflammatory response is crucial for the recovery of skeletal muscle following ischemia. Therefore, macrophage-based therapeutic targets need to be explored for ischemic disease. In the current study, we found that the mRNA levels of scavenger receptor A1 (*Sr-a1*) were elevated in patients with critical limb ischemia, based on an analysis of the Gene Expression Omnibus data. We then investigated the role and underlying mechanisms of macrophage SR-A1 in a mouse hindlimb ischemia (HLI) model. Compared with the *Sr-a1*^fl/fl^ mice, the *Lyz*^Cre/+^/*Sr-a1*^flox/flox^ (*Sr-a1*^ΔMΦ^) mice showed significantly reduced laser Doppler blood flow in the ischemic limb on day seven after HLI. Consistently, histological analysis revealed that the ischemic limb of the *Sr-a1*^ΔMΦ^ mice exhibited more severe and prolonged necrotic morphology, inflammation, fibrosis, decreased vessel density, and delayed regeneration than that of the control *Sr-a1*^fl/fl^ mice. Furthermore, restoring wild-type myeloid cells to the *Sr-a1* knockout mice effectively improved the Doppler perfusion in the ischemic limb and mitigated skeletal muscle damage seven days after HLI. Consistent with these *in vivo* findings, co-cultivating macrophages with the mouse myoblast cell line C2C12 revealed that the *Sr-a1*^−/−^ bone marrow macrophages significantly inhibited myoblast differentiation *in vitro*. Mechanistically, SR-A1 enhanced the skeletal muscle regeneration in response to HLI by inhibiting oncostatin M production *via* suppression of the NF-κB signaling activation. These findings indicate that SR-A1 may be a promising candidate protein to improve tissue repair and regeneration in peripheral ischemic arterial disease.

## Introduction

Peripheral artery disease (PAD) is a cardiovascular condition characterized by partial or complete obstruction of one or more peripheral arteries. These arterial blockages restrict the blood supply to organs outside the heart, with the limbs being the most common site of PAD manifestation. Globally, over 200 million individuals suffer from PAD, and its incidence increases significantly with age, being more prevalent in males^[1]^. Critical limb ischemia (CLI) is a severe form of PAD related to ischemic rest pain, walking impairment, and/or a high incidence of permanent limb tissue loss, such as gangrene or ulcerations. These devastating outcomes have attracted increasing interest in optimal management of limb salvage, including cell therapies aimed at improving limb perfusion and muscle regeneration. Unfortunately, PAD remains a complex disease because of the limited benefits and effectiveness of current medical strategies^[2]^. Blood perfusion to the lower limbs in patients with PAD heavily depends on the degree of vascular remodeling (from revascularization) in the ischemic limb muscle. However, PAD not only damages blood vessels but also severely impacts skeletal muscles, which are critical to limb function because of their reliance on sufficient oxygen and energy delivery^[3]^. Therefore, fundamental studies on the cellular and molecular biology of ischemic injury-induced muscle regeneration are needed to develop new treatment options for skeletal muscle salvage in the low extremities of PAD patients, independent of limb perfusion.

Skeletal muscle, the most abundant tissue in the body, possesses a remarkable regenerative capacity. The repair process is supported by a dedicated population of myogenic precursor (satellite) cells, known as muscle stem cells (MuSCs). These stem cells remain in a quiescent state until tissue damage triggers their activation, promoting self-renewal and differentiation to regenerate the surrounding damaged tissues^[4]^. The regenerative potential of MuSCs is supported by various cell types within the MuSC niche, including macrophages, which play a crucial role in facilitating tissue repair^[5]^.

During skeletal muscle regeneration in response to injury, monocytes and macrophages interact with satellite cells to orchestrate muscle repair. Muscle regeneration is achieved through the infiltration of monocyte-derived macrophages that activate myoblasts^[6]^. Ly6C^high^ macrophages release pro-inflammatory cytokines that promote myoblast proliferation but inhibit differentiation, whereas Ly6C^low^ macrophages release anti-inflammatory cytokines that restrain myoblast proliferation but stimulate differentiation and fusion^[7]^. Although the well-established role of macrophages in skeletal muscle regeneration is known, whether and how macrophages regulate the limb tissue repair in response to an ischemic insult in the context of CLI remains to be elucidated.

Scavenger receptor A1 (SR-A1), a subclass of scavenger receptors, is a trimeric transmembrane glycoprotein and is primarily expressed in macrophages^[8]^. Studies have shown that SR-A1 predominantly recognizes and clears a wide range of polymeric anions presented in circulation and tissues. It plays a pivotal role in various biological processes such as foam cell formation, cell adhesion, cell proliferation, and host defense^[9]^. For example, SR-A1 attenuated mice cardiomyocyte necrosis induced by the left anterior descending coronary artery ligation by suppressing macrophage polarization toward a skewed M1 phenotype^[10]^. Additionally, SR-A1 has been found to promote the osteogenic differentiation of bone marrow stem cells *in vitro*^[11]^.

However, the role of SR-A1 in peripheral artery disease remains poorly understood. In the current study, we investigated whether and how macrophage-derived SR-A1 played a role in tissue damage and regeneration in the context of ischemic limb injury.

## Materials and methods

### Animals

Mice with global or conditional knockout of *Sr-a1* were described previously^[12]^. The *Lyz*^Cre/+^/*Sr-a1*^flox/flox^ (*Sr-a1*^ΔMΦ^) mice were generated by crossing *Lyz*^Cre/+^ mice with the conditional *Sr-a1*-allele (*Sr-a1*^fl/fl^) mice. Wild-type C57BL/6J mice were obtained from GemPharmatech Co. Ltd., Nanjing, China. All animal study protocols were reviewed and approved by the Animal Ethical and Welfare Committee of Nanjing Medical University. Mice were maintained in a facility free from specific pathogens, provided with continuous access to food and water, and subjected to 12-h light/12-h dark cycles.

### Hindlimb ischemia (HLI) model

Male mice, aged 8–12 weeks, were used to establish a HLI model. Under aseptic conditions, the mice were anesthetized with intraperitoneal injection of ketamine hydrochloride (100 mg/kg) and xylazine (5 mg/kg), and then underwent lower limb surgery. The surgical procedure involved blunt dissection of the femoral artery, followed by ligation at both the proximal end of the femoral artery and the distal segment of the saphenous artery. The section of the femoral artery between the ligatures was then removed^[13]^. In contrast, the sham-operated mice received similar surgical manipulation without any artery ligation or removal. Mice were sacrificed on days 1, 3, 7, and 14 after the induction of HLI, and tissues were harvested from both limbs for subsequent analyses.

### Laser Doppler blood flow imaging

At various time points (before, 6 h, 1 day, 3 days, 7 days, and 14 days after HLI), mice were anesthetized using 2% isoflurane *via* inhalation and were positioned on a heated platform maintaining a body temperature of approximately 37 (± 0.5) ℃ to minimize the anesthesia's effect on the blood flow imaging. The Moor Laser Doppler Imager (MoorLDI2, Moor Instruments, Axminster, Devon, UK) was used to examine the blood flow in the lower limbs of the mice. The collected data were processed using MoorLDI image processing software (version 6.1), and the results were expressed as the ratio of blood flow between the ischemic hind paw and the nonischemic contralateral control paw.

### Necrosis score

On the 7th day following surgery, we performed a semiquantitative evaluation to assess tissue damage and functional recovery. Necrosis in the ischemic limb was evaluated according to a previously established scale: a score of 0 indicated no necrosis, 1 indicated necrosis confined to the toes, 2 indicated necrosis extending to the dorsum pedis, 3 indicated necrosis extending to the crus, and 4 was assigned for necrosis reaching the mid-tibia or encompassing the entire limb^[14]^.

### Bone marrow transplantation

Four-week-old male recipient mice, including wild-type or *Sr-a1*-deficient genotypes, were irradiated with eight Gy. Subsequently, five million bone marrow cells from wild-type or *Sr-a1* knockout mice were injected intravenously *via* the tail vein. Hindlimb ischemia was initiated four weeks after bone marrow reconstitution.

### Histological analysis

Gastrocnemius muscles were preserved in 4% paraformaldehyde for 24 h, and then dehydrated, embedded in paraffin, and sectioned into 5-μm slices. Alternatively, for cryosections, the muscles were dehydrated in 30% sucrose before being frozen and cut into 5-μm sections using optimal cutting temperature compounds. The hematoxylin and eosin staining was used to assess necrosis and count regenerating myofibers, while Masson's trichrome staining was used to assess total collagen deposition.

In preparation for immunofluorescence staining, the slides were brought to room temperature and fixed using ice-cold 4% paraformaldehyde for 10 min, and then were permeabilized using 0.5% Triton X-100 in PBS for 10 min. Blocking was performed using 5% normal donkey serum in PBS at room temperature for 30 min, followed by incubation with primary antibody at 4 ℃ overnight. The primary antibodies used were anti-SR-A1 (1∶200; Cat. #NBP1-00092, Novus Biologicals, Littleton, CO, USA), anti-CD3 (1∶100; Cat. #100244, BioLegend, San Diego, CA, USA), anti-lymphocyte antigen 6 family member G (Ly6G; 1∶100, Cat. #127602, BioLegend), anti-galectin 3 (also known as MAC2; 1∶200, Cat. #CL8942AP, CEDARLANE, CA, USA), anti-laminin (1∶200, Cat. #ab11575, Abcam, Cambridge, UK), anti-embryonic myosin heavychain (eMyHC; 1∶50, Cat. #AB_528358, DSHB, Seattle, WA, USA), anti-CD31 (1∶100, Cat. #550274, BD Biosciences, San Jose, CA, USA), and anti-Desmin (1∶400, Cat. #ab32362, Abcam). Simultaneously, isotype-matched controls were used as negative controls (***Supplementary Fig. 1***, available online). After primary staining, sections were washed in PBS and incubated with Alexa 488- (1∶3000, product #A32766, Invitrogen, Carlsbad, CA, USA) or Alexa 546- (1∶3000, product #A10040, Invitrogen) conjugated secondary antibodies at room temperature for 1 h, and then were counterstained with DAPI (product #0100-20, Southern Biotech, Birmingham, AL, USA). Images captured using a Zeiss fluorescence microscope (Oberkochen, Deutschland) were analyzed in a blinded manner using Image-Pro Plus 6.0 software.

### Cell culture

Mouse C2C12 myoblasts were acquired from the National Collection of Authenticated Cell Cultures, and their potential for myogenic differentiation was validated by immunofluorescence assays for specific myogenic markers, such as myogenin and MyoD. The C2C12 cells were maintained in DMEM high glucose growth medium, supplemented with 10% FBS, 1% penicillin/streptomycin, and 2 mmol/L L-glutamine. For cell differentiation, the growth medium was switched to a differentiation medium containing high-glucose DMEM supplemented with either 5% FBS or 2% horse serum, along with 1% penicillin/streptomycin, and 2 mmol/L L-glutamine, facilitating the fusion into myotubes. The differentiation medium was refreshed every other day. All cell-based experiments were performed in a controlled environment, specifically in a humidified incubator at 37 ℃ with 5% CO_2_.

Bone marrow-derived macrophages (BMDMs) were obtained from the bone marrow of male and female mice aged 4–8 weeks as described previously^[15]^. The harvested cells were cultured in RPMI 1640 medium supplemented with 10% FBS, 100 U/mL penicillin, 100 µg/mL streptomycin, and 0.2 μg/mL macrophage colony-stimulating factor. The culturing process lasted for 7 to 9 days to promote macrophage differentiation.

### RNA-seq analysis

The hindlimb muscles were harvested from both the *Sr-a1*^ΔMΦ^ and *Sr-a1*^fl/fl^ mice 3 days after the HLI surgery. The collected muscles were then minced and subjected to enzymatic digestion using a buffer containing 0.1% collagenase, 0.05% BSA, and 0.25% trypsin. This mixture was incubated at 37 ℃ for 20 min with gentle agitation. Serum was added to halt the enzymatic reaction. Then the tissue debris was eliminated through cell strainers. The digested tissues were centrifuged at 500 *g* for 5 min. After the use of erythrocyte lysate and PBS, cells were resuspended in PBS containing 2% FBS, and incubated for 30 min with APC-Cy7-anti-CD45 (1∶200, product #557659, BD Pharmingen [San Diego, CA, USA]), PE-anti-CD11b (1∶200, product #2394478, Invitrogen), and BV711-anti-F4/80 (1∶200, product #123147, BioLegend) at room temperature. Subsequently, cells were washed and resuspended in cold PBS before FACS analysis or flow sorting by a flow cytometer (FACSVerse, BD Biosciences).

Transcriptomic analysis was performed using RNA sequencing on total RNA extracted from mouse macrophages in ischemic muscle tissues of both the *Sr-a1*^ΔMΦ^ mice and *Sr-a1*^fl/fl^ mice. The library construction, sequencing processes, and data analysis were carried out by BGI Genomics Co. Ltd (Beijing, China).

### *In vitro* C2C12 cocultures with BMDMs

To prepare a BMDM-conditioned medium, 2 × 10^6^ BMDMs were incubated in DMEM supplemented with 10% FBS and 1% antibiotics for 48 h. Separately, C2C12 myoblasts were maintained in growth medium for no more than 10 passages to ensure viability and functionality. For the differentiation assay, C2C12 cells (6 × 10^4^ per well) were seeded and cultured in a growth medium for 24 h. Then the growth medium was switched to a differentiation medium to promote myotube formation. On the second day of myoblast differentiation, the medium was supplemented with the BMDM-conditioned differentiation medium for an additional three days to support myotube differentiation.

### Quantitative reverse transcription-PCR (qRT-PCR) analysis

Total RNA was isolated from both tissues and cells by using RNAiso Plus (TaKaRa, Japan). One microgram of RNA was reversely transcribed to cDNA. Real-time PCR was then performed using the ABI Prism 7000 PCR system. The relative expression levels of genes were calculated using the comparative cycle threshold method (2^−ΔΔCt^) and normalized to a housekeeping gene. Specific oligonucleotide primers used for target genes are shown in ***Supplementary Table 1*** (available online).

### Western blotting

Proteins were isolated from tissues or cells using RIPA buffer (product #P0013B, Beyotime, Shanghai, China) supplemented with a protease inhibitor cocktail (product #4693132001, Roche, Basel, Switzerland). The concentration of the extracted proteins was determined using a BCA protein assay (product #23227, Thermo Scientific, Waltham, MA, USA). An equal amount of total proteins was loaded into each lane for electrophoresis, and then transferred onto polyvinylidene difluoride membranes. After blocking with 5% bovine serum albumin, the membranes were sequentially incubated with primary and secondary antibodies. Chemiluminescence signals of separated proteins were semi-quantified using ImageJ software. Antibodies used for Western blotting are detailed in ***Supplementary Data*** (available online).

### Statistical analysis

Student's *t*-tests were used for comparisons between two groups, and one-way or two-way ANOVA followed by Tukey's multiple comparison tests for multiple group comparisons. These analyses were performed using GraphPad Prism version 8.0 software. All data points collected in the current study were included in statistical analyses. All data were presented as mean ± standard error of the mean, and a *P*-value of less than 0.05 was considered to indicate statistical significance.

## Results

### SR-A1 was upregulated in macrophages of the ischemic hindlimb

**Figure 1:**
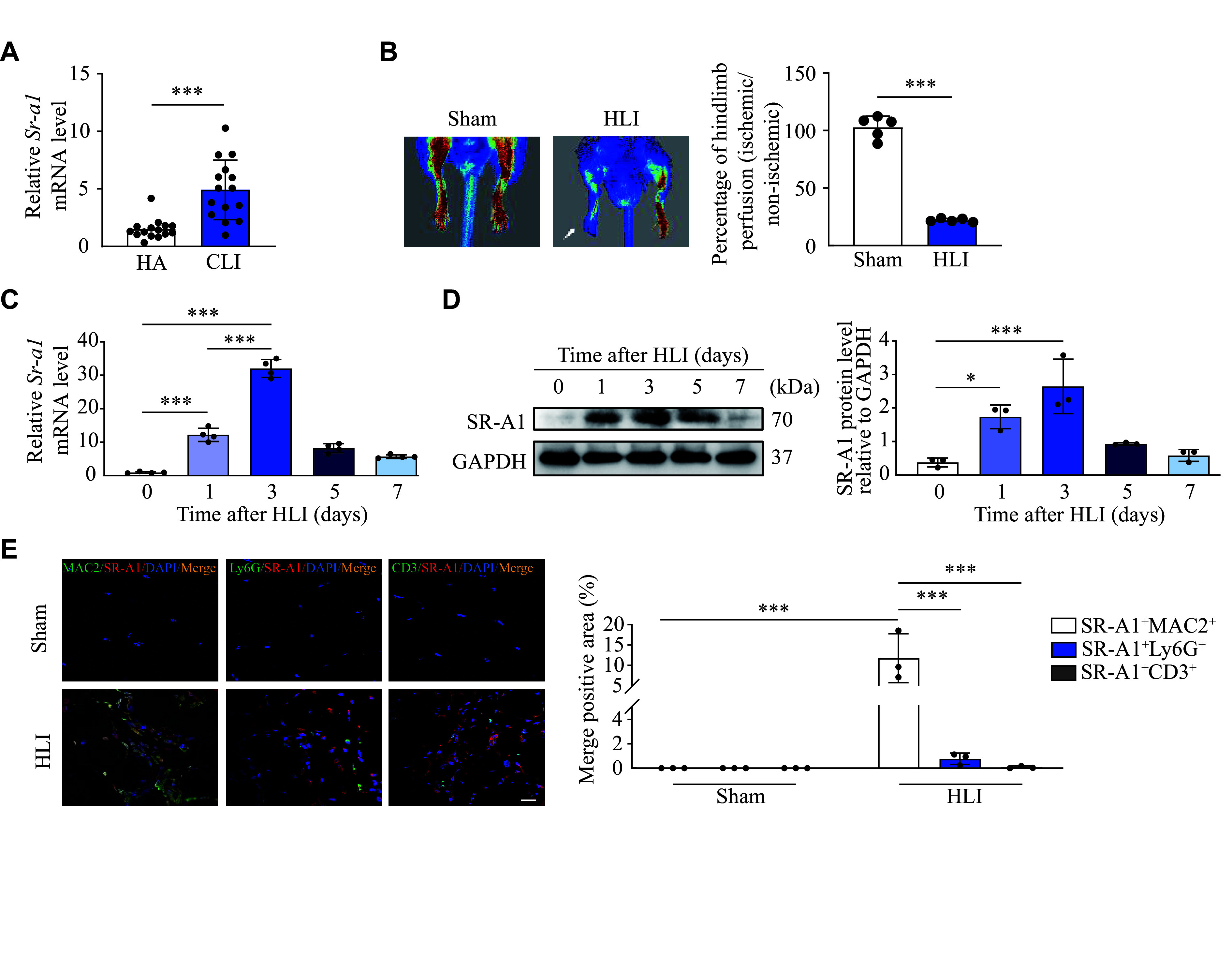
SR-A1 was upregulated in macrophages of the ischemic hindlimb. A: The mRNA levels of *Sr-a1* in HA and CLI patients (data from GSE120642). *n* = 15. B: Schematic representation of femoral artery ligation performed on mice (left) and quantification of hindlimb perfusion in sham and HLI groups (right). *n* = 5. C and D: The *Sr-a1* mRNA levels (C; *n* = 4) and SR-A1 protein levels (D; *n* = 3) in the gastrocnemius tissues of wild-type mice at the indicated time points after the HLI surgery were detected by qRT-PCR and Western blotting, respectively. E: Representative immunofluorescence images of a cross-section of ischemic muscles stained with MAC2 (the marker for macrophages, green), Ly6G (the marker for neutrophils, green), CD3 (the marker for T lymphocytes, green), SR-A1 (red), and DAPI (blue). The merged positive areas (SR-A1^+^MAC2^+^, SR-A1^+^Ly6G^+^, and SR-A1^+^CD3^+^) were quantified. Scale bar: 20 μm. *n* = 3. All data are presented as mean ± standard error of the mean. ^*^P < 0.05 and ^***^P < 0.001 by two-tailed Student's *t*-test (A and B), one-way ANOVA with Tukey's multiple comparisons test (C–D), and two-way ANOVA followed by Tukey's multiple comparisons test (E). Abbreviations: HA, healthy adult; CLI, critical limb ischemia; HLI, hindlimb ischemia; SR-A1, scavenger receptor A1.

To assess whether SR-A1 contributes to muscle recovery, we analyzed the public RNA-seq data from CLI patients (GSE120642). Interestingly, the mRNA levels of *Sr-a1* were significantly upregulated in CLI patients compared with healthy adults (***Fig. 1A***). We also examined the expression of SR-A1 in muscles by generating a murine ischemic hindlimb surgery, in which the femoral artery was excised and the blood flow of crural right limb muscle was reduced by more than 70% (***Fig. 1B***). The qRT-PCR analysis showed increased mRNA levels of *Sr-a1* in the ischemic hindlimb tissues (soleus and gastrocnemius) within one day after ischemia, peaking on the third day and returning to baseline levels on days 5 to 7 (***Fig. 1C***). These observations were corroborated by Western blotting analysis of SR-A1 expression in the ischemic hindlimb tissues post femoral artery excision (***Fig. 1D***). Additionally, immunofluorescence staining revealed the macrophage infiltration and SR-A1 upregulation in the gastrocnemius muscles of the HLI mice compared with the sham mice. Of note, SR-A1 was preferentially co-localized with macrophages instead of neutrophils or T lymphocytes (***Fig. 1E***). The dynamic changes in macrophage SR-A1 suggest its involvement in the pathophysiology of hindlimb ischemia.

### SR-A1 depletion in myeloid cells restrained perfusion recovery after HLI

**Figure 2:**
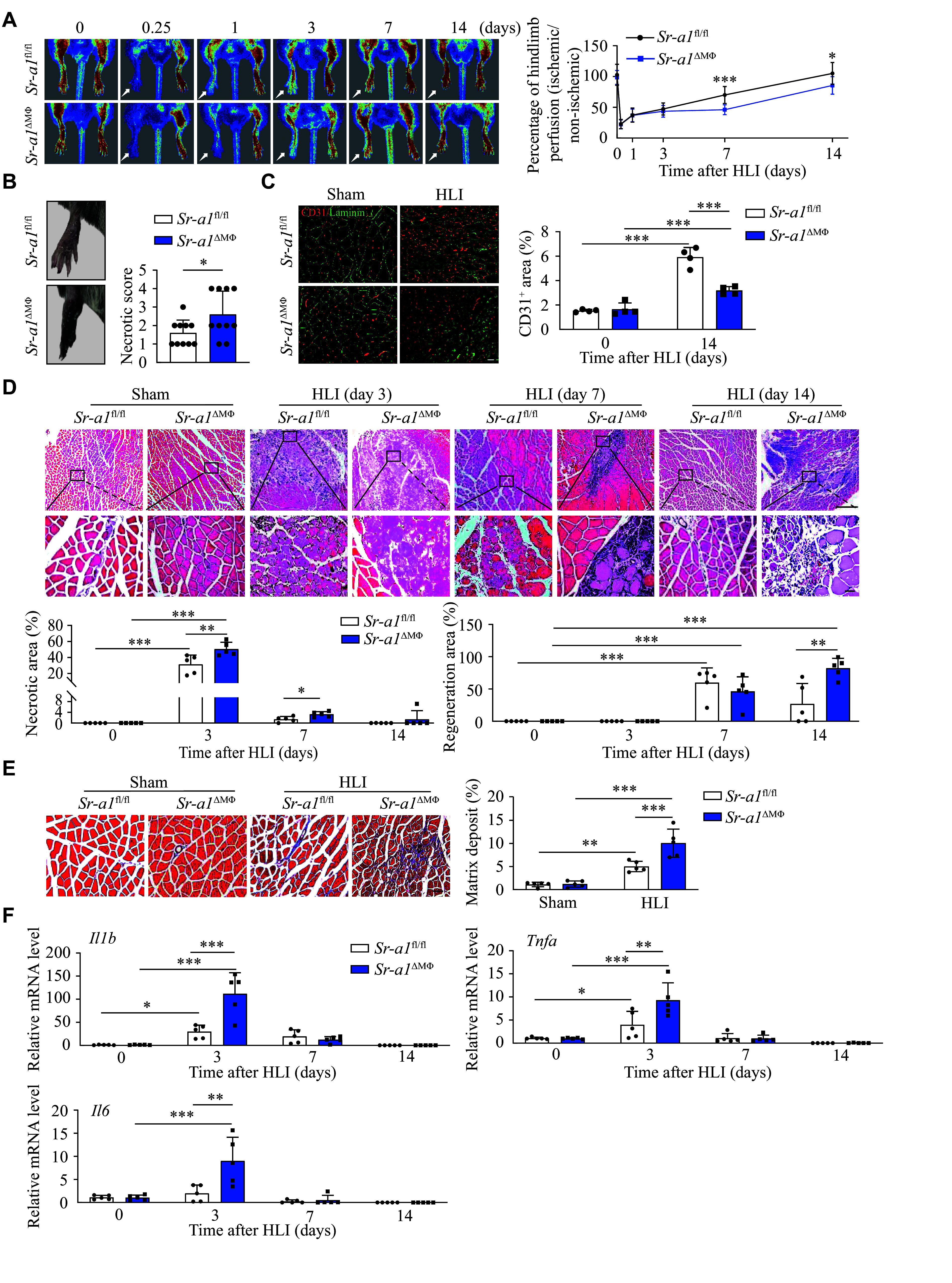
SR-A1 depletion in myeloid cells impeded muscle repair. A: Representative images and quantitative analysis of the hindlimb blood perfusion in the *Sr-a1*^fl/fl^ and *Sr-a1*^ΔMΦ^ mice at the indicated time points after HLI. *n* = 8–10. B: Limb necrosis scores at 7 days after HLI in the *Sr-a1*^fl/fl^ and *Sr-a1*^ΔMΦ^ mice. *n* = 10. C: Immunofluorescence staining and quantitative analysis of CD31 in the *Sr-a1*^fl/fl^ and *Sr-a1*^ΔMΦ^ mice at 14 days after HLI. CD31 (red) and laminin (green). Scale bar: 20 μm. *n* = 4. D: Hematoxylin and eosin staining of gastrocnemius muscles in the *Sr-a1*^fl/fl^ and *Sr-a1*^ΔMΦ^ mice at the indicated time points after HLI, with the necrosis and regeneration areas quantified. Scale bar: 100 μm (upper) or 20 μm (lower). *n* = 5. E: Masson staining of gastrocnemius muscles in the *Sr-a1*^fl/fl^ and *Sr-a1*^ΔMΦ^ mice at 14 days after HLI, with the ratio of interstitial fibrosis quantified. Scale bar: 20 μm. *n* = 5. F: Relative mRNA levels of *Il1b*, *Tnfa*, and *Il6* in gastrocnemius tissues of the *Sr-a1*^fl/fl^ and *Sr-a1*^ΔMΦ^ mice at the indicated time points after HLI. *n* = 5. All data are presented as mean ± standard error of the mean. ^*^P < 0.05, ^**^P < 0.01, and ^***^P < 0.001 by two-tailed Student's *t*-test (B) and two-way ANOVA followed by Tukey's multiple comparisons test (A and C–F). Abbreviation: HLI, hindlimb ischemia.

Next, we used mice with myeloid-specific ablation of *Sr-a1* (*Sr-a1*^fl/fl^*Lyz*^Cre+^, *Sr-a1*^ΔMΦ^) to investigate the role of macrophage SR-A1 in the HLI model. After femoral artery ligation, blood flow recovery was significantly impaired, and ischemia and limb damage were further deteriorated in the *Sr-a1*^ΔMΦ^ mice compared with the *Sr-a1*^fl/fl^ mice (***Fig. 2A***). The *Sr-a1*^ΔMΦ^ mice showed more severe toe necrosis than the *Sr-a1*^fl/fl^ mice (***Fig. 2B***).

Angiogenesis plays a crucial role in the recovery of perfusion after ischemic events. To assess the extent of angiogenesis, CD31 immunostaining is commonly employed as an index of capillary formation. Consistent with the quantitative measure of blood flow in the ischemic limb shown in ***Fig. 2A***, vascular density was lower in the *Sr-a1*^ΔMΦ^ mice at 14 days after HLI, compared with that of the *Sr-a1*^fl/fl^ mice (***Fig. 2C***). Additionally, SR-A1 deficiency in myeloid cells significantly aggravated cell necrosis in the ischemic gastrocnemius muscles and delayed the regeneration of skeletal muscles after injury (***Fig. 2D***). Specifically, 14 days after muscle injury, the control mice were basically in a state of complete regeneration, whereas the mice lacking SR-A1 still had a large number of cells in the regeneration stage, showing a larger regeneration area than the control mice. Moreover, SR-A1 deficiency in myeloid cells significantly increased the matrix deposit in the ischemic hindlimb at 14 days after HLI (***Fig. 2E***). The mRNA levels of *Il1β*, *Tnfa*, and *Il6* in the ischemic limb of the *Sr-a1*^ΔMΦ^ mice at 3 days after femoral artery excision were significantly higher than those of the *Sr-a1*^fl/fl^ mice (***Fig. 2F***). Collectively, these findings indicate that the ablation of SR-A1 in myeloid cells may exacerbate ischemia-induced muscle injury.

### SR-A1 expression sustained proper regenerative myogenesis after HLI

**Figure 3:**
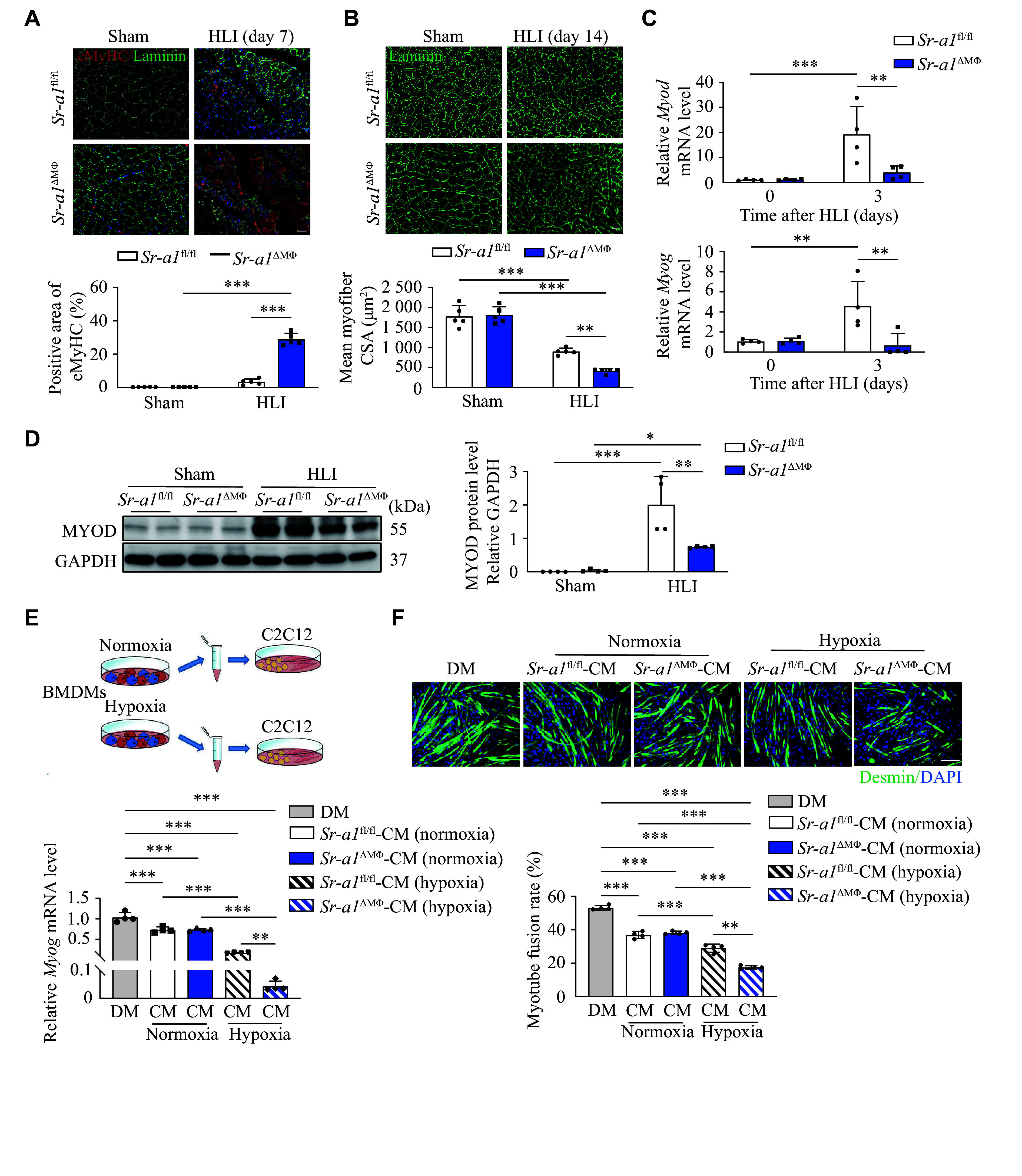
SR-A1 expression sustained proper regenerative myogenesis after HLI. A: Immunofluorescence staining and quantitative analysis of eMyHC expression in the *Sr-a1*^fl/fl^ and *Sr-a1*^ΔMΦ^ mice at 7 days after HLI. eMyHC (red) and laminin (green). Scale bar: 20 μm. *n* = 5. B: Immunostaining images of laminin (green) and mean cross-sectional area (CSA) of muscle fibers at 14 days after HLI. Scale bar: 20 μm. *n* = 5. C: Relative *Myod* and *Myog* mRNA levels in gastrocnemius muscles of the *Sr-a1*^fl/fl^ and *Sr-a1*^ΔMΦ^ mice at 3 days after HLI. *n* = 4. D: Western blotting and quantitative analysis of MYOD protein levels in ischemic gastrocnemius tissues of the *Sr-a1*^fl/fl^ and *Sr-a1*^ΔMΦ^ mice at 3 days after HLI. *n* = 4. E and F: C2C12 cells were first cultured with differentiation medium (DM) for 24 h, then co-cultured with conditioned medium (CM; the BMDM supernatant under hypoxic or normoxic stimulation conditions) for 48 h. Relative *Myog* mRNA levels were detected by qRT-PCR (E; *n* = 4). Desmin^+^ myotubes were detected by immunofluorescence staining (F; *n* = 4). Desmin (green) and DAPI (blue). Scale bar: 50 μm. All data are presented as mean ± standard error of the mean. ^*^P < 0.05, ^**^P < 0.01, and ^***^P < 0.001 by two-way ANOVA followed by Tukey's multiple comparisons test (A–D) and one-way ANOVA with Tukey's multiple comparisons test (E and F). Abbreviations: HLI, hindlimb ischemia; eMyHC, embryonic myosin heavy chain.

To investigate the effects of SR-A1 deficiency on regenerative myogenesis, we performed immunofluorescence staining for eMyHC, a marker of early regeneration in skeletal muscle, in both *Sr-a1*^fl/fl^ and *Sr-a1*^ΔMΦ^ muscles. The results showed that the *Sr-a1*^ΔMΦ^ mice had a larger number of eMyHC^+^ muscle fibers in their muscles at 7 days post-injury than the *Sr-a1*^fl/fl^ mice (***Fig. 3A***), indicating that the *Sr-a1*^fl/fl^ mice had reached a late stage of regeneration, whereas the *Sr-a1*^ΔMΦ^ mice were still in an early stage of regeneration. This result suggests that SR-A1 deficiency may slow down skeletal regeneration in the HLI mice.

Analysis of myofiber cross-sectional area showed that *Sr-a1*^ΔMΦ^ myofibers were smaller than *Sr-a1*^fl/fl^ myofibers at 14 days after HLI (***Fig. 3B***). We further detected the mRNA levels of myogenic regulatory factors *Myod* and *Myog* in total muscles of both *Sr-a1*^fl/fl^ and *Sr-a1*^ΔMΦ^ mice at 3 days post-injury, relative to uninjured control muscles. The expression levels of *Myod* and *Myog* were induced in the *Sr-a1*^fl/fl^ injured muscle, consistent with active regeneration. However, reduced levels of *Myod* and *Myog* were observed in the *Sr-a1*^ΔMΦ^ ischemic muscles, compared with those of the *Sr-a1*^fl/fl^ ischemic muscles (***Fig. 3C***). These observations were corroborated by Western blotting, which showed that the protein levels of MYOD were lower in the damaged muscles of the *Sr-a1*^ΔMΦ^ mice than in the *Sr-a1*^fl/fl^ mice at 3 days after injury (***Fig. 3D***).

To further demontrate the role of SR-A1 in macrophage-mediated skeletal muscle regeneration, we isolated BMDMs from both *Sr-a1*^fl/fl^ and *Sr-a1*^ΔMΦ^ mice and co-cultured them with C2C12, an immortalized mouse myoblast cell line. As expected, co-culturing with macrophages from *Sr-a1*^fl/fl^ and *Sr-a1*^ΔMΦ^ mice both weakened the differentiation ability of C2C12 cells. Under hypoxic conditions, the differentiation ability of C2C12 cells co-cultured with macrophages from *Sr-a1*^ΔMΦ^ mice was further suppressed (***Fig. 3E***). Immunofluorescence staining of C2C12 myotubes also showed consistent results (***Fig. 3F***). These results were not reliant on direct physical interaction between BMDMs and C2C12, demonstrating that macrophage SR-A1 may regulate myoblast differentiation *via* a secreted factor.

Collectively, these findings imply a delayed regenerative process in the *Sr-a1*^ΔMΦ^ mice, suggesting that SR-A1 expression played a crucial role in efficient muscle repair in adult mice.

### Oncostatin M was up-regulated in SR-A1-deficient macrophages

**Figure 4:**
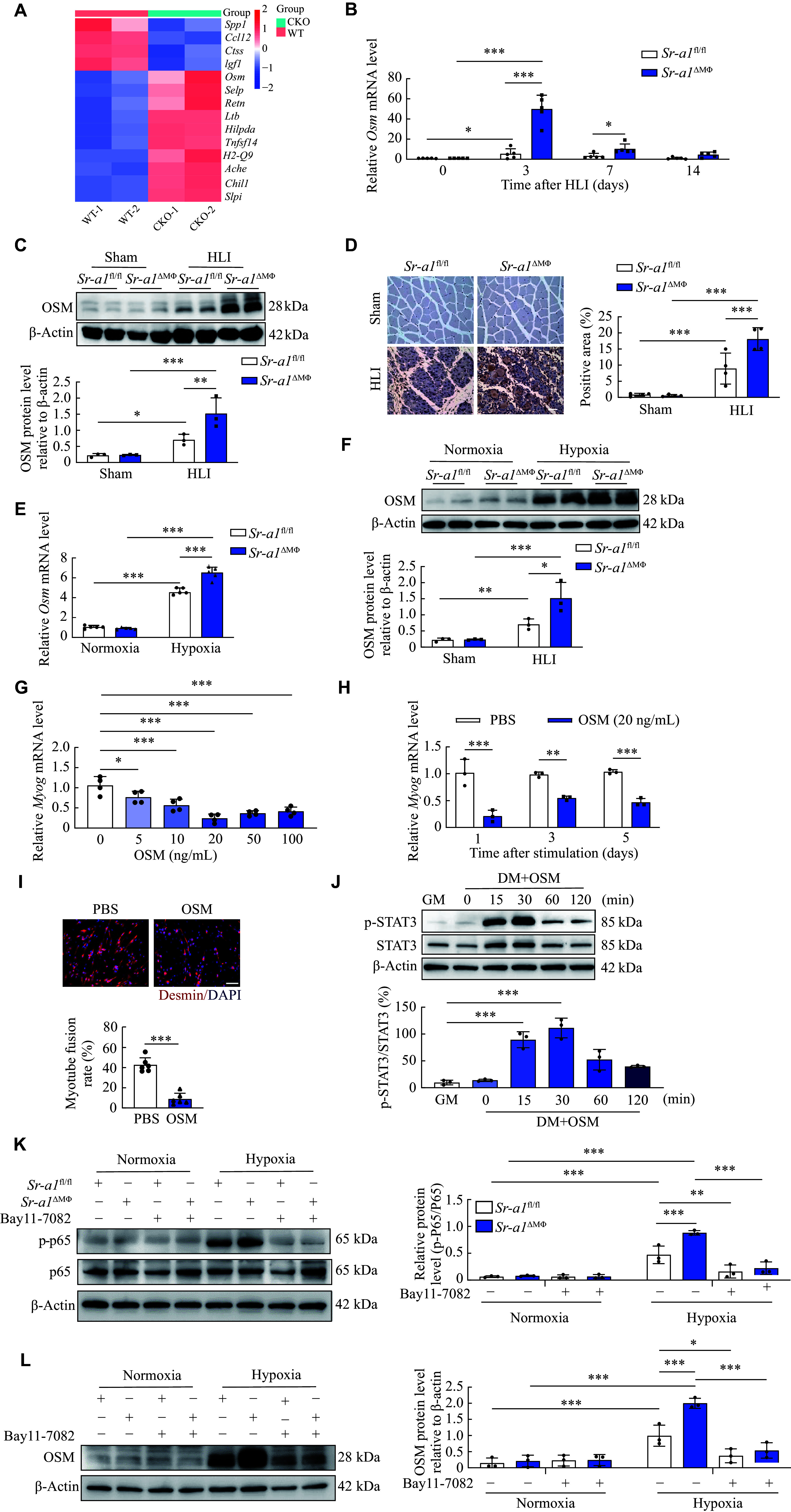
Oncostatin M was up-regulated in SR-A1-deficient macrophages. A: Heatmap of RNA-seq analysis of the ischemic gastrocnemius tissues of the *Sr-a1*^fl/fl^ (WT) and *Sr-a1*^ΔMΦ^ (CKO) mice at 3 days after HLI. B: Relative mRNA levels of *Osm* in the gastrocnemius tissues of the *Sr-a1*^fl/fl^ and *Sr-a1*^ΔMΦ^ mice at the indicated time points after HLI. *n* = 5. C and D: OSM protein levels in ischemic skeletal muscle tissues of the *Sr-a1*^fl/fl^ and *Sr-a1*^ΔMΦ^ mice at 3 days after HLI were detected by Western blotting (C; *n* = 3) and immunohistochemical staining (D; *n* = 4), respectively. Scale bar: 20 μm. E and F: The *Osm* mRNA levels (E; *n* = 5) and OSM protein levels (F; *n* = 3) in BMDMs from the *Sr-a1*^fl/fl^ and *Sr-a1*^ΔMΦ^ mice treated under normoxia or hypoxia condition for 2 h were detected by qRT-PCR and Western blotting, respectively. G: Relative *Myog* mRNA levels in C2C12 cells after differentiation induced by OSM at the indicated concentrations. *n* = 4. H: *Myog* mRNA levels in C2C12 cells treated with OSM (20 ng/mL) for 1, 3, and 5 days. *n* = 3. I: Immunofluorescence staining and quantitative analysis of myotube fusion rate (desmin^+^ myotube) in C2C12 cells. Desmin (red) and DAPI (blue). Scale bar: 50 μm. *n* = 6. J: Western blotting and quantitative analysis of phospho-STAT3 and total STAT3 protein levels in C2C12 cells treated with OSM for the indicated time periods. *n* = 3. K and L: BMDMs from the *Sr-a1*^fl/fl^ and *Sr-a1*^ΔMΦ^ mice were treated with or without the NF-κB pathway inhibitor BAY 11-7082 (10 μmol/L) for 1 h, and then were treated under normoxia or hypoxia for 2 h. Phosphorylated P65 (p-P65), total P65 (K), and OSM (L) protein levels were analyzed by Western blotting. *n* = 3. All data are presented as mean ± standard error of the mean. ^*^P < 0.05, ^**^P < 0.01, and ^***^P < 0.001 by two-way ANOVA followed by Tukey's multiple comparisons test (B–F, H, K, and L), two-tailed Student's *t*-test (I), and one-way ANOVA with Tukey's multiple comparisons test (G and J). Abbreviations: HLI, hindlimb ischemia; OSM, oncostatin M.

To assess how SR-A1 affects hindlimb ischemia, we performed RNA sequencing analysis of CD45^+^CD11b^+^F4/80^+^ macrophages isolated from the ischemic gastrocnemius muscle tissues of both *Sr-a1*^fl/fl^ and *Sr-a1*^ΔMΦ^ mice at 3 days after HLI (***Fig. 4A***). Myeloid *Sr-a1* knockout resulted in a significant alteration in gene expression, with 115 genes downregulated and 716 genes upregulated. Notably, oncostatin M (*Osm*), a pleiotropic cytokine, was the most upregulated gene after the loss of SR-A1 (***Fig. 4A***). Moreover, the mRNA levels of *Osm* in the ischemic muscles at 3, 7, and 14 days after HLI were significantly higher in the SR-A1-deficient mice than in the control mice. However, the deletion of *Sr-a1* did not change *Osm* mRNA levels in skeletal muscle under basal conditions (***Fig. 4B***). The Western blotting analysis showed that protein levels of OSM were higher in ischemic skeletal muscle tissues from the *Sr-a1*^ΔMΦ^ mice than in the *Sr-a1*^fl/fl^ mice (***Fig. 4C***), while immunohistochemical staining further demonstrated these changes (***Fig. 4D***). Plasma levels of OSM were also increased after HLI, and the loss of SR-A1 enhanced this effect (***Supplementary Fig. 2***, available online). Additionally, the expression levels of OSM were significantly upregulated in BMDMs exposed to 1% oxygen for 2 h compared with those in normoxia, which were obtained from both *Sr-a1*^fl/fl^ and *Sr-a1*^ΔMΦ^ mice, and SR-A1 deficiency further enhanced this stimulatory effect of hypoxia (***Fig. 4E*** and ***4F***). OSM treatment significantly inhibited *Myog* mRNA levels in a dose-dependent manner (***Fig. 4G***), and the most significant inhibitory effect was observed at 1 day of stimulation with 20 ng/mL OSM in C2C12 cells (***Fig. 4H***). Immunofluorescence staining confirmed that OSM significantly inhibited the myoblast fusion (***Fig. 4I***).

Studies have indicated that the Janus kinase-signal transducer and activator of transcription 3 (JAK1-STAT3) signaling pathway, sensitized by OSM, plays a crucial role in the regulation of myoblast maintenance and differentiation^[16]^. This pathway may have a significant effect on the regeneration and repair processes of the muscle. In the current study, we found that OSM significantly induced phosphorylation of STAT3 in C2C12 cells, with the most significant upregulation of STAT3 phosphorylation 30 min after treatment (***Fig. 4J***). Notably, there was also a significant increase in the phosphorylation of p65 in BMDMs obtained from the *Sr-a1*1^ΔMΦ^ mice following 30 min of hypoxic stimulation. This increase was repressed by Bay 11-7082, a nuclear factor kappa-B (NF-κB) inhibitor (***Fig. 4K***). Furthermore, treatment with Bay 11-7082 also blocked the OSM expression response to hypoxic exposure in BMDMs from both *Sr-a1*^fl/fl^ and *Sr-a1*^ΔMΦ^ mice (***Fig. 4L***). Collectively, these data demonstrate that SR-A1 deficiency upregulates the OSM expression in the HLI muscles.

### Macrophage SR-A1 promoted recovery from limb ischemia

**Figure 5:**
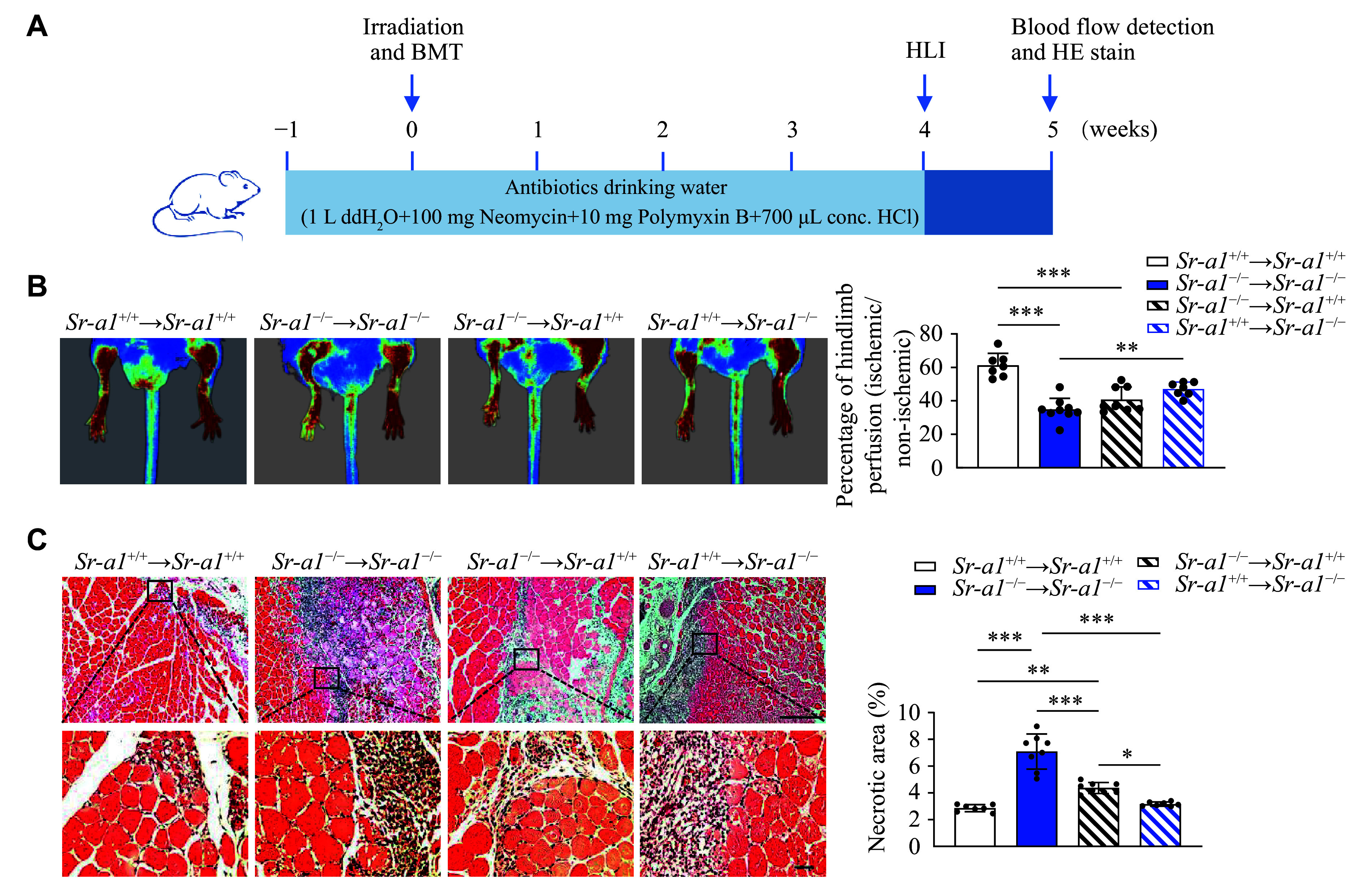
Macrophage SR-A1 exerted a protective effect in HLI. A: Schematic representation of the procedure for bone marrow transplantation (BMT). B: Representative images and quantitative analysis of blood perfusion in hindlimbs of four groups of the HLI modeling mice at 7 days after HLI. *n* = 7–9. C: Hematoxylin and eosin (HE) staining of gastrocnemius muscles in four groups of the HLI modeling mice at 7 days after HLI. Scale bar: 100 μm (upper) and 20 μm (lower). *n* = 7–8. All data are presented as mean ± standard error of the mean. ^*^P < 0.05, ^**^P < 0.01, and ^***^P < 0.001 by one-way ANOVA with Tukey's multiple comparisons test. Abbreviations: HLI, hindlimb ischemia; ddH_2_O, double distilled water; conc. HCl, concentrated hydrochloric acid.

Finally, to investigate the prophylactic significance of the macrophage SR-A1 in critical hindlimb ischemia, we performed bone marrow transplantation experiments in both wild-type and global *Sr-a1* knockout mice. This approach allowed us to assess how bone marrow-derived cells, particularly those expressing SR-A1, influence the recovery and repair processes in ischemic limbs (***Fig. 5A***). As shown in ***Fig. 5B***, the bone marrow transplantation from the *Sr-a1*^−/−^ mice to the *Sr-a1*^+/+^ mice aggravated skeletal muscle injury induced by femoral artery excision, whereas the restoration of SR-A1 in myeloid cells through bone marrow transplantation significantly reduced hindlimb ischemia after femoral artery excision in the SR-A1-deficient mice. At day 7, mice transplanted with the *Sr-a1* knockout bone marrow displayed reduced hindlimb perfusion and increased necrotic areas, compared with those transplanted with the *Sr-a1*^+/+^ bone marrow, regardless of the recipient mice genotype (***Fig. 5B*** and ***5C***). These data demonstrate that macrophage SR-A1 promotes the recovery of perfusion and the preservation of ischemic hindlimb.

## Discussion

The current study is the first to specifically investigate the role of macrophage SR-A1 in the skeletal muscle regeneration in response to limb ischemia. Expression levels of SR-A1 were upregulated in the ischemic hindlimb of PAD patients and during the early stage of the HLI mice induced by femoral artery ligation. Myeloid SR-A1 deficiency resulted in an aggravated inflammatory response and a delayed muscle regeneration. Early after ischemia, morphological changes induced by SR-A1 deficiency in crural muscles were accompanied by increased expression of pro-inflammatory markers. Furthermore, the restoration of myeloid SR-A1 by bone marrow transplantation enhanced vascular perfusion and improved the skeletal muscle regeneration in response to ischemia. Additionally, we identified that SR-A1 deficiency impaired the skeletal muscle regeneration during HLI by upregulating the OSM expression *via* activation of the NF-κB signaling. These findings indicate that the SR-A1/OSM axis may be a potential therapeutic target for PAD.

It is well established that following acute muscle injury, inflammatory cells rapidly infiltrate the damaged tissues. Neutrophils are the first responders, typically infiltrating within hours, followed by the accumulation of monocytes and macrophages, peaking around the third day^[17]^. Subsequently, macrophage numbers gradually decline, with only a small population remaining in the muscle by the seventh day^[18]^. Macrophages play a central role in muscle regeneration, and their interactions with satellite cells, endothelial cells, and fibro-adipogenic progenitors directly influence the quality and efficiency of the process^[19–20]^.

Macrophage-derived glutamine promotes satellite cell proliferation and differentiation, thereby facilitating the recovery of injured or aging muscles^[21–22]^. M2-polarized macrophages modulate endothelial cell function by promoting vascular neogenesis in muscle tissues by the secretion of VEGFA^[21–22]^. In the current study, we found that OSM inhibited myoblast differentiation, consistent with the actions of certain cytokines. Additionally, resident macrophages rarely participate in regeneration. OSM is a cytokine belonging to the interleukin-6 family, characterized as a secreted glycoprotein primarily produced by immune cells such as monocytes and macrophages.

OSM has been found to promote the quiescence of muscle satellite cells and inhibit their myogenic differentiation in response to cardiotoxin injury *in vitro*^[23]^. OSM primarily inhibits myoblast differentiation by activating the JAK1/STATs pathway, and the prolonged expression of OSM in muscles disrupts the regenerative process^[16,24]^. Similarly, the triggering receptor expressed on myeloid cells 2 (TREM2)^+^ macrophages also negatively regulates hair follicle growth to maintain hair follicle stem cell quiescence^[25]^. Additionally, reparative macrophages coordinate muscle regeneration, specifically through the production of OSM, by regulating the coupling of myogenesis and angiogenesis^[26]^. Studies suggest that muscle fibers may produce OSM, inducing reversible cell cycle exits in muscle stem cells and promoting their quiescence^[23]^. Consistent with our findings, single-cell RNA sequencing revealed abundant OSM transcripts produced by monocytes and macrophages were found 0.5 days after cardiotoxin-induced hindlimb muscle injury by single-cell RNA sequencing; moreover, OSM, together with IL-1β and TNF-α, strongly promotes muscle stem cell regeneration and skeletal muscle repair^[27]^. In the current study, through sequencing, we observed the increased transcription of OSM in *Sr-a1* knockout macrophages, and we are focusing on understanding how SR-A1 regulates OSM production.

As a vital class of scavenger receptors on macrophages, SR-A1 exerts a profound influence on the functionality and characteristics of these cells. The pathways influenced by SR-A1 comprise, at the very least, receptors, associated signaling molecules, and regulatory elements. SR-A1 resides within the cell membrane, specifically in coated pits and lipid rafts, where its interaction with ligands activates various signaling pathways, including protein kinase C (PKC), heterotrimeric G proteins of the Gi/o family, mitogen-activated protein kinase (MAPK), and NF-κB, among others^[28]^. The overall or bone marrow-specific ablation of SR-A1 inhibits the proliferation of cardiac resident reparative macrophages, exacerbating doxorubicin-induced cardiomyopathy^[12]^. Deficiency in SR-A1 not only results in macrophage dysfunction but also leads to their abnormal accumulation, causing an increase in apoptotic cells within the diseased aortic wall^[29]^. Additionally, SR-A1 influences efferocytosis by activating the TYRO3 protein tyrosine kinase (TYRO3) signaling in macrophages, thereby contributing to the development of aortic dissection induced by beta-aminopropionitrile (BAPN)^[29]^. The current study demonstrates that the deficiency of SR-A1 may increase the OSM transcription by activating the NF-κB subunit p65. Studies have shown that OSM may be transcriptionally regulated by NF-κB in osteoblasts^[30]^ and by AP-1 in macrophages^[31]^. The current study provides some further evidence for SR-A1 modulation of the NF-κB pathway and, for the first time, identifies its direct regulatory role in OSM production.

We acknowledge the potential limitations of the current study. The effect of macrophage inflammation on the initial differentiation of muscle stem cells in both human chronic limb-threatening ischemia and animal models remains a fascinating subject^[20]^. It is important to note that the CLI condition in PAD patients is featured by chronic limb-threatening ischemia, whereas animal models typically represent regeneration responses to acute ischemia. Therefore, other cellular behaviors and molecular pathways, including dystrophic muscles, extracellular matrix deposition, and scar formation, as well as unknown factors generated during the long-term ischemic phase, may also play a significant role in the failure of muscle regeneration. Our current study provides some new perspectives on the role of macrophages in tissue homeostasis and regeneration.

Taken together, the current work demonstrates that macrophage SR-A1 promotes recovery from hindlimb ischemia by repressing inflammatory response. SR-A1 deficiency exacerbates the OSM production induced by ischemic injury, leading to delayed skeletal muscle regeneration in mouse HLI. Our findings indicate that SR-A1 may be a promising therapeutic target for PAD.

## SUPPLEMENTARY DATA

Supplementary data to this article can be found online.
