# Supplementary material for: Macrophage scavenger receptor A1 promotes skeletal muscle regeneration after hindlimb ischemia
Source: J Biomed Res. 2024 May 29;39(1):23–35. doi: 10.7555/JBR.38.20240117 (PMC11873590; doi:10.7555/JBR.38.20240117)
Supplement: Supplementary file 1 — Supplementary data to this article can be found online. [file jbr-39-1-23-S1.pdf]

Available online at www.jbr-pub.org.cn

Open Access at PubMed Central

Journal of Biomedical Research, 2025 39(1): 1–3

Supplementary Data

JBR

Macrophage scavenger receptor A1 promotes skeletal muscle regeneration after hindlimb ischemia

Siying Wang[1,△], Saiya Wang[1,△], Wenhan Cai[1], Jie Wang[1], Jianan Huang[1], Qing Yang[1], Hui Bai[1,2], Bin Jiang[1,2], Jingjing Ben[1,2], Hanwen Zhang[1,2], Xudong Zhu[1,2], Xiaoyu Li[1,✉], Qi Chen[1,2,✉]

[1]*Department of Pathophysiology, Key Laboratory of Targeted Intervention of Cardiovascular Disease and Molecular Intervention, Collaborative Innovation Center for Cardiovascular Disease Translational Medicine, Nanjing Medical University, Nanjing, Jiangsu 211166, China;*

[2]*The Affiliated Suzhou Hospital of Nanjing Medical University, Suzhou Municipal Hospital, Gusu School, Nanjing Medical University, Nanjing, Jiangsu 211166, China.*

Supplemental materials and methods

Antibodies used in Western blotting

The antibodies used were as follows: anti-SR-A1 (1 ∶ 1 000; Cat. #NBP1-00092, NOVUS Biologicals, Littleton, CO, USA); Anti-OSM (1 ∶ 1 000; Cat. #PA5-79775, Invitrogen, Carlsbad, CA, USA); anti-β-actin (1 ∶ 3 000; Cat. #66009-1-Ig, Proteintech, Wuhan, China); anti-GAPDH (1 ∶ 3 000; Cat. #kc-5G4, Kang ChengTech, Shanghai, China); anti-MyoD (1 ∶ 500; Cat. #sc-377460, Santa Cruz Biotechnology, Santa Cruz, CA, USA); anti-STAT3 (1 ∶ 1 000; Cat. #12640S, Cell Signaling Technology, Danvers, MA, USA); anti-phospho-STAT3 (Tyr705; 1 ∶ 1 000; Cat. #9145S, Cell Signaling Technology); anti-p65 (1 ∶ 1 000; Cat. #8242S, Cell Signaling Technology); anti-phospho-p65 (1 ∶ 1 000; Cat. #3033S, Cell Signaling Technology); HRP-conjugated anti-mouse (1 ∶ 3 000; Cat. #SN134, Best Biological, Nanjing, China); HRP-conjugated anti-rabbit (1 ∶ 3 000; Cat. #SN133, Best Biological).

Negative control in immunostaining studies

To determine that the binding of the primary

Supplementary Table 1 **Mouse oligonucleotide primer sequences for quantitative reverse transcription-PCR**

Genes	Forward primer (5′-3′)	Reverse primer (5′-3′)
Il1b	TGTCTTGGCCGAGGACTAAGG	TGGGCTGGACTGTTTCTAATGC
Tnfa	ACGGCATGGATCTCAAAGAC	AGATAGCAAATCGGCTGACG
Il6	CTGCAAGAGACTTCCATCCAG	AGTGGTATAGACAGGTCTGTTGG
Sr-a1	AGAGGGCTTACTGGACAAACTG	GGCTTTCCTGGTGCTCCTG
Myod	CACTGTAGTAGGCGGTGTC	CTACAGTGGCGACTCAGAT
Myog	AAGGCAACAGACATATCCTC	CAGTACATTGAGCGCCTAC
Osm	GCACGGGCCAGAGTACCAGGAC	CTGGTGTTGTAGTGGACCGTGAG
Gapdh	CCATTTGCAGTGGCAAAG	CACCCCATTTGATGTTAGTG

△These authors contributed equally to this work.

✉Corresponding authors: Qi Chen and Xiaoyu Li, Department of Pathophysiology, Key Laboratory of Targeted Intervention of Cardiovascular Disease and Molecular Intervention, Collaborative Innovation Center for Cardiovascular Disease Translational Medicine, 101 Longmian Avenue, Jiangning District, Nanjing, Jiangsu 211166, China. E-mails: qichen@njmu.edu.cn (Chen) and xyli@njmu.edu.cn (Li).

Received: 24 April 2024; Revised: 11 May 2024; Accepted: 15 May 2024; Published online: 29 May 2024

CLC number: R543, Document code: A

The authors reported no conflict of interests.

This is an open access article under the Creative Commons Attribution (CC BY 4.0) license, which permits others to distribute, remix, adapt and build upon this work, for commercial use, provided the original work is properly cited.

© 2025 by Journal of Biomedical Research.

https://doi.org/10.7555/JBR.38.20240117

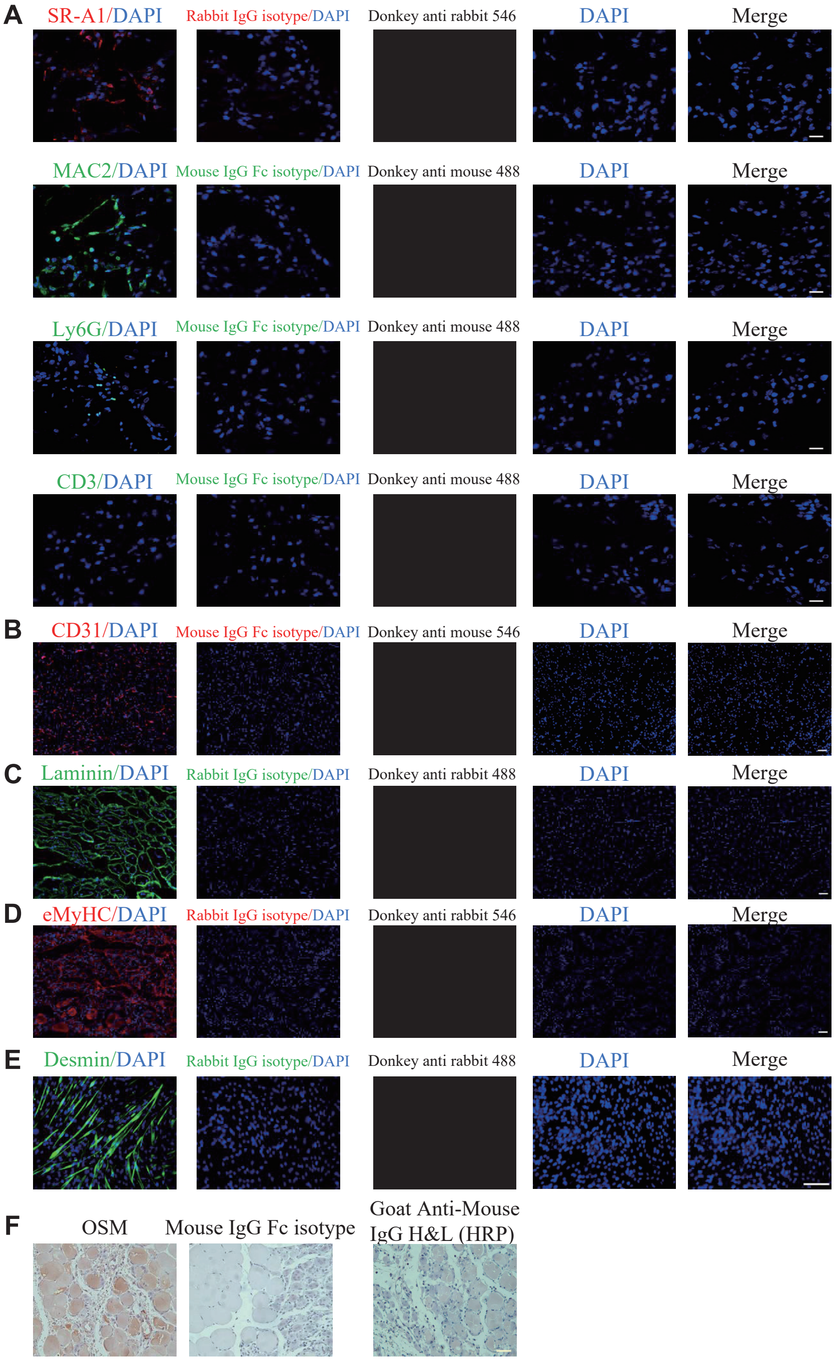

Supplementary Fig. 1 **Negative control in immunostaining studies.** A: Negative controls for ***Fig. 1E***. Immunofluorescence staining of SR-A1 (red), MAC2 (green), Ly6G (green), CD3 (green), and DAPI (blue), and their negative controls using fluorescence-labeled isotype-matched immunoglobulins to replace primary antibodies or fluorescence-labeled isotype-matched secondary antibodies only. Scale bar: 20 µm. B: Negative controls for ***Fig. 2C***. Immunofluorescence staining of CD31 (red) and DAPI (blue), and their negative controls using fluorescence-labeled isotype-matched immunoglobulins to replace primary antibodies or fluorescence-labeled isotype-matched secondary antibodies only. Scale bar: 20 µm. C: Negative controls for ***Fig. 2C***, ***Fig. 3A*** and ***3B***. Immunofluorescence staining of Laminin (green) and DAPI (blue), and their negative controls using fluorescence-labeled isotype-matched immunoglobulins to replace primary antibodies or fluorescence-labeled isotype-matched secondary antibodies only. Scale bar: 20 µm. D: Negative controls for ***Fig. 3A***. Immunofluorescence staining of eMyHC (red) and DAPI (blue), and their negative controls using fluorescence-labeled isotype-matched immunoglobulins to replace primary antibodies or fluorescence-labeled isotype-matched secondary antibodies only. Scale bar: 20 µm. E: Negative controls for ***Fig. 3F***. Immunofluorescence staining of Desmin (green) and DAPI (blue), and their negative controls using fluorescence-labeled isotype-matched immunoglobulins to replace primary antibodies or fluorescence-labeled isotype-matched secondary antibodies only. Scale bar: 50 µm. F: Negative controls for ***Fig. 4D***. Immunohistochemical staining of OSM and its negative controls using fluorescence-labeled isotype-matched immunoglobulin to replace primary antibody or fluorescence-labeled isotype-matched secondary antibody only. Scale bar: 20 µm. Abbreviations: OSM, oncostatin M; eMyHC, embryonic myosin heavychain.

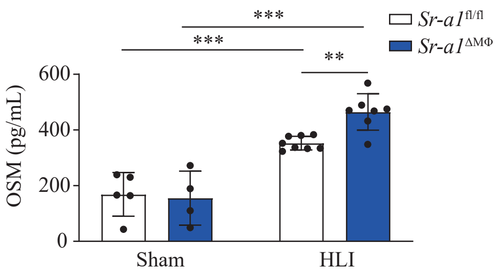

Supplementary Fig. 2 **The OSM levels in the plasma.** The plasma OSM levels of *Sr-a1*$^{fl/fl}$ and *Sr-a1*$^{\Delta M\Phi}$ mice at 0 and 3 days after HLI. $n = 4–8$. All data are presented as mean ± standard error of the mean. $^{**}P < 0.01$ and $^{***}P < 0.001$ by two-way ANOVA followed by Tukey's multiple comparisons test. Abbreviations: OSM, oncostatin M; HLI, hindlimb ischemia.

antibody is specific and not a non-specific Fc receptor or interaction with other proteins, we used the same species source, the same subtype, the same dose, and the same immunoglobulin and subtype of the primary antibody (isotype control). To exclude non-specific binding of secondary antibodies,we set up a group of samples without the addition of primary antibodies and directly added secondary antibodies as a negative control.

Mouse IgG Fc isotype control (1 ∶ 500; Cat. #31205) and Rabbit IgG isotype control (1 ∶ 500; Cat. #31235) were purchased from Invitrogen (Carlsbad, CA, USA).

Enzyme-linked immunosorbent assay (ELISA)

Murine blood samples were collected from the *Sr-a1*$^{fl/fl}$ and *Sr-a1*$^{\Delta M\Phi}$ mice before and three days after HLI. Plasma levels of OSM were determined by using the ELISA kits (Cat. #CSB-E04697) from CUSABIO Life Sciences (Wuhan, China) according to the manufacturer's instructions.
